# Association of schizophrenia onset age and white matter integrity with treatment effect of D-cycloserine: a randomized placebo-controlled double-blind crossover study

**DOI:** 10.1186/s12888-017-1410-3

**Published:** 2017-07-12

**Authors:** Kazuo Takiguchi, Akihito Uezato, Michio Itasaka, Hidenori Atsuta, Kenji Narushima, Naoki Yamamoto, Akeo Kurumaji, Makoto Tomita, Kazunari Oshima, Kosaku Shoda, Mai Tamaru, Masahito Nakataki, Mitsutoshi Okazaki, Sayuri Ishiwata, Yasuyoshi Ishiwata, Masato Yasuhara, Kunimasa Arima, Tetsuro Ohmori, Toru Nishikawa

**Affiliations:** 1 Department of Psychiatry and Behavioral Sciences, Graduate School of Medical and Dental Sciences, Tokyo Medical and Dental University, 1-5-45, Yushima, Bunkyo-ku, Tokyo, 113-8519 Japan; 2 Haryugaoka Hospital, Tensyoudan 11, Otsukimachi, Koriyama-shi, Fukushima, 963-0201 Japan; 3 Psychiatry Department, Tokyo Metropolitan Tama Medical Center, 2-8-29 Musashidai, Fuchu-shi, Tokyo, 183-8524 Japan; 4 Clinical Research Center, Medical Hospital of Tokyo Medical and Dental University, 1-5-45, Yushima, Bunkyo-ku, Tokyo, 113-8519 Japan; 5 Ohmiya Kousei Hospital, Katayanagi 1, Minuma-ku, Saitama-shi, Saitama, 337-0024 Japan; 6 Department of Psychiatry, Institute of Biomedical Sciences, Tokushima University Graduate School, 3-18-15, Kuramoto, Tokushima, 770-8503 Japan; 7 Department of Psychiatry, National Center Hospital of Neurology and Psychiatry, 4-1-1, Ogawa-Higashi, Kodaira, Tokyo, 187-8551 Japan; 8 Department of Hospital Pharmacy, Tokyo Medical and Dental University, 1-5-45, Yushima, Bunkyo-ku, Tokyo, 113-8519 Japan

**Keywords:** Schizophrenia, D-cycloserine, Glutamate, MR diffusion tensor imaging, Randomized, Placebo-controlled, Double-blind, Crossover

## Abstract

**Background:**

It has been reported that drugs which promote the *N*-Methyl-D-aspartate-type glutamate receptor function by stimulating the glycine modulatory site in the receptor improve negative symptoms and cognitive dysfunction in schizophrenia patients being treated with antipsychotic drugs.

**Methods:**

We performed a placebo-controlled double-blind crossover study involving 41 schizophrenia patients in which D-cycloserine 50 mg/day was added-on, and the influence of the onset age and association with white matter integrity on MR diffusion tensor imaging were investigated for the first time. The patients were evaluated using the Positive and Negative Syndrome Scale (PANSS), Scale for the Assessment of Negative Symptoms (SANS), Brief Assessment of Cognition in Schizophrenia (BACS), and other scales.

**Results:**

D-cycloserine did not improve positive or negative symptoms or cognitive dysfunction in schizophrenia. The investigation in consideration of the onset age suggests that D-cycloserine may aggravate negative symptoms of early-onset schizophrenia. The better treatment effect of D-cycloserine on BACS was observed when the white matter integrity of the sagittal stratum/ cingulum/fornix stria terminalis/genu of corpus callosum/external capsule was higher, and the better treatment effect on PANSS general psychopathology (PANSS-G) was observed when the white matter integrity of the splenium of corpus callosum was higher. In contrast, the better treatment effect of D-cycloserine on PANSS-G and SANS-IV were observed when the white matter integrity of the posterior thalamic radiation (left) was lower.

**Conclusion:**

It was suggested that response to D-cycloserine is influenced by the onset age and white matter integrity.

**Trial registration:**

UMIN Clinical Trials Registry (number UMIN000000468). Registered 18 August 2006

## Background

Since *N*-Methyl-D-aspartate-type glutamate receptor (NMDA receptor) blockers, including phencyclidine and ketamine, were clarified to induce schizophrenia-like positive and negative symptoms and cognitive dysfunction in proportion to the potency, dysfunction of glutamate neurotransmission through the NMDA receptor in schizophrenia is assumed [1, 2]. Accordingly, improvement of intractable schizophrenia symptoms by enhancing the NMDA receptor is expected, and it has been reported that drugs promoting the NMDA receptor function by stimulating the glycine modulatory site in this receptor improved negative symptoms and cognitive dysfunction in schizophrenia patients being treated with antipsychotic drugs [3, 4].

D-cycloserine (DCS) was clinically used as an antituberculosis drug, but its partial agonist action on the NMDA receptor glycine modulatory site was newly reported in 1989 [5]. Since then, clinical studies of DCS aiming at improvement of schizophrenia symptoms by activating the NMDA receptor have been performed [6–20]. Compared with other usable agonists of the glycine modulatory site, the dose of DCS needed is lower because it readily passes though the blood-brain barrier, and the dose for psycho-neurologic disease is about 10–30% of that for tuberculosis treatment, being readily clinically applicable. In several previous clinical studies, the optimum enhancement of the NMDA receptor function can be expected at about 50 mg/day. Although improvement of negative symptoms and cognitive dysfunction was reported, the results of the clinical studies were not consistent. It is possible that this inconsistency is due to heterogeneity of the clinical profile among patients analyzed. For example, early-onset schizophrenia is considered a more severe form with a poor prognosis [21, 22] and the difference in the onset age may influence response to treatment, but these were not investigated in previous studies. In addition, recent imaging studies suggested that differences in the brain structure influence the treatment outcome (reviewed in [23]). To address the influence of illness onset and the impact of altered brain structure, in this study, a placebo-controlled double-blind study of DCS was performed involving schizophrenia patients, and the efficacy and its association with the onset age and white matter integrity determined by diffusion tensor imaging were investigated.

## Methods

### Study design

A randomized, placebo-controlled, double-blind, crossover design trial was performed at multicenters (Tokyo Medical and Dental University (TMDU), National Center of Neurology and Psychiatry, Tokushima University, Haryugaoka Hospital, Ohmiya Kousei Hospital). We described this clinical trial according to the CONSORT 2010 guidelines. A 2-week pre-observation period was set before initiation of drug administration to confirm eligibility of the subjects, followed by registration and randomized allocation. The subsequent 6, 3, and 6 weeks were established as the first, washout, and second phases, respectively. The rater, medical care staff, subjects, and their families did not know the allocated test drug, and DCS (50 mg/day) and placebo (PCB) were prepared with capsules indistinguishable from the appearance. To confirm the participants’ adherence to the study protocol, blood levels of DCS were measured by high performance liquid chromatography (HPLC) with electrochemical detection in the pre-observation period and at the end of the first and second phases. The subjects continued taking drugs that they usually took by the same administration method and dose throughout the study period.

### Inclusion and exclusion criteria

The subjects were patients aged 20 years or older and below 70 years with schizophrenia meeting the diagnostic criteria of DSM-IV classification regardless of being in- or outpatients. Patients who received ‘psychotherapy and formulated cognitive behavioral therapy excluding supportive psychotherapy’ within 12 weeks, those diagnosed with substance abuse (alcohol or drug) or substance dependence within 24 weeks based on the DSM-IV classification, those who received electroconvulsive therapy within 12 weeks, those with suicide attempt within 24 weeks, those with a past medical history of or complication by convulsive disease (epilepsy etc.), those with a past medical history of hypersensitivity to DCS and lactose, and those with severe organic disorder of the brain and physical symptoms were excluded. Participants who smoked were included but no individual information was obtained. Many participants were on benzodiazepines and they were maintained on the same dosage throughout the study. No participant was on clozapine.

### Psychometry

One rater evaluated changes in neurologic manifestations using the Positive and Negative Syndrome Scale (PANSS) and Scale for the Assessment of Negative Symptoms (SANS) every 2 weeks from initiation of the first and second phases. The scores before initiation of each phase were used as baseline scores for each phase. Evaluations were also made before initiation and after completion of the first phase and after completion of the second phase using Brief Assessment of Cognition in Schizophrenia (BACS), Emotional Intelligence Scale (EQS), Global Assessment of Functioning (GAF), and the Japanese version of the Calgary Depression Scale for Schizophrenia (JCDSS). In addition, extrapyramidal symptoms were evaluated using the Drug Induced Extra-Pyramidal Symptoms Scale (DIEPSS) and Abnormal Involuntary Movement Scale (AIMS). The scores before initiation of the first phase were used as baseline scores for both the first and second phase. Clinical testing of blood and urine were also performed to continuously observe the occurrence of adverse events to evaluate the safety.

### Image acquisition

Magnetic resonance (MR) diffusion tensor imaging (DTI) was performed in the pre-observation period at TMDU. Imaging data were acquired on the 3.0 T MRI scanner (3.0 T Signa HDxt, GE Healthcare Japan). Diffusion tensor images were constructed of 2 sets of diffusion weight axial slice images (30 directions at b-value = 1000, and a b-value = 0 s/mm^2^, slice thickness = 3.0 mm, slice gap = 0, repetition time = 14,000 ms, echo time = 72.5, matrix 128 × 128 pixels, voxel resolution = 1.875 × 1.875 × 3, Flip angle = 90°, the number of slices scanned ranged 1600–1800.).

### Tract based spatial statistics (TBSS)

To reveal the white matter abnormality in schizophrenia, statistical analysis was performed using the FSL (FMRIB Software Library, FMRIB, Oxford, UK) software package including FDT (FMRIB’s Diffusion Toolbox). First, imaging data were collected for the distortion and head motion, calculated by using the “eddy correct” function. A brain mask was made from the b = 0 image to use brain extraction of tensor image, and then fractional anisotropy (FA), mean diffusivity (MD), axial diffusivity (AD), radial diffusivity (RD) maps were calculated by using the “dtifit” function to fit tensor model. All parameters at each voxel were performed by voxel-wise statistics as follows.

Tract based spatial statistics (TBSS) were applied on the common pseudoanatomical skeleton in which all subjects’ FA data were aligned to FMRIB58_FA standard –space image, using nonlinear registration tool [24]. And then atlas based region of interest (ROI) analysis was performed by using JHU DTI 81 labels atlas. This atlas included the 48 white matter tracts such as corpus callosum, fornix, and internal capsule. The voxel mean FA of these tracts was calculated in each subject.

### Statistical analysis

Statistical analysis was performed using the IBM SPSS Statistics software (version 23.0). The significance level was set at *p* < 0.05. Multiple comparison was not taken into consideration.

#### Patient characteristics

Difference between the DCS-first and PCB-first groups on age, age at onset, age at starting therapy, duration of illness, education, or CPZ equivalents were examined by Mann-Whitney U-test.

#### Effect of treatment by time

Three subscales of PANSS (positive symptoms (P), negative symptoms (N), and general psychopathology (G)) and 7 subscales of SANS (I, II, III, IV, V, summary score, and total score) were analyzed by 2-way repeated-measures of analysis of covariance (RM-ANCOVA) constructed of within-subject factors of treatment (DCS/PCB) and time (baseline, week 2, week 4, and week 6) and covariates of treatment order. Seven subscales of BACS (verbal memory (VM), digit sequencing task (DS), token motor task (TM), symbolic coding (SC), verbal fluency of category (VFC), verbal fluency of letter (VFL), and tower of London (TOL)) and 3 subscales of EQS (intrapersonal, interpersonal, and situational domains) were also analyzed using RM-ANCOVA with a within-subject factor of time (baseline and week 6). Furthermore, GAF, JCDSS, DIEPSS, and AIMS were also analyzed using RM-ANCOVA with a within-subject factor of time (baseline and week 6). If Mauchly’s sphericity test was significant, then the Greenhouse-Geisser correction was applied.

#### Correlation between DCS-effect and DTI white matter integrity

To investigate whether or not the white matter integrity influences response to DCS, we first introduced a parameter ‘DCS-effect’ which was defined by scores at DCS phase subtracted by those at PCB phase. DCS-effect is a value to indicate the net effect of DCS excluding placebo effect. Correlations between the tract FA values of the 48 brain regions and DCS-effects on PANSS, SANS, BACS, and EQS were analyzed using Pearson’s correlation coefficient. Significance level for the correlation analysis was set *p* < 0.01. Multiple comparison was not taken into consideration.

### Secondary analysis: Onset age and DCS-effect

To investigate whether or not the onset age influences response to DCS, patients were stratified by onset ages of <18 years (early-onset schizophrenia, EOS) or 18 years or older (non-early-onset schizophrenia, non-EOS), according to the generally accepted threshold for EOS [25]. Of note, one non-EOS patient was traditionally-defined late-onset (45 years or older) schizophrenia.

EOS (*n* = 6) and non-EOS (*n* = 30) were compared using multivariate analysis of covariance (MANCOVA) regarding the DCS-effects on subscales of PANSS (P, N and G), SANS (I-V, summary and total), BACS (VM, DS, TM, SC, VFC, VFL, and TOL) and EQS (intrapersonal, interpersonal, and situational domains) as dependent variables, onset age (EOS vs. non-EOS) as an independent variable, and treatment order as a covariate.

### Sample preparation and high-performance liquid chromatography (HPLC) with electrochemical detection

The concentrations of DCS were quantitated by HPLC with electrochemical detection according to the method of Takahashi et al. [26]. For this assay, the plasma samples provided by the patients were homogenized in 2 volumes of 10% trichloroacetic acid. The supernatants resulting from centrifugation of the homogenate at 14,500 g for 20 min at 4 °C were neutralized by 2 M imidazole and stored at −80 °C until derivatization. An aliquot of each sample was derivatized with o-phthaldialdehyde (OPA) (nakalai tesque, Japan) for 2 min at room temperature. The derivatized sample was immediately applied to the HPLC system (HTEC-500; EICOM, Japan) and then analyzed on a on a SC-5ODS (φ3.0 × 150 mm) with a pre-column. The separation column was operated at the constant flow rate of 0.5 ml/min at 30 °C. Mobile phase was 50% 50 mM phosphate buffer solution (pH 2.8) containing 5 mg/L disodium-ethylenediaminetetraacetic acid (EDTA) and 50% methanol. DCS was eluted in the mobile phase within 10 min.

The presence of substantial plasma concentrations of DCS after the actual drug periods, but not the placebo periods, in each patient was verified.

## Results

### Patient characteristics

**Fig. 1:**
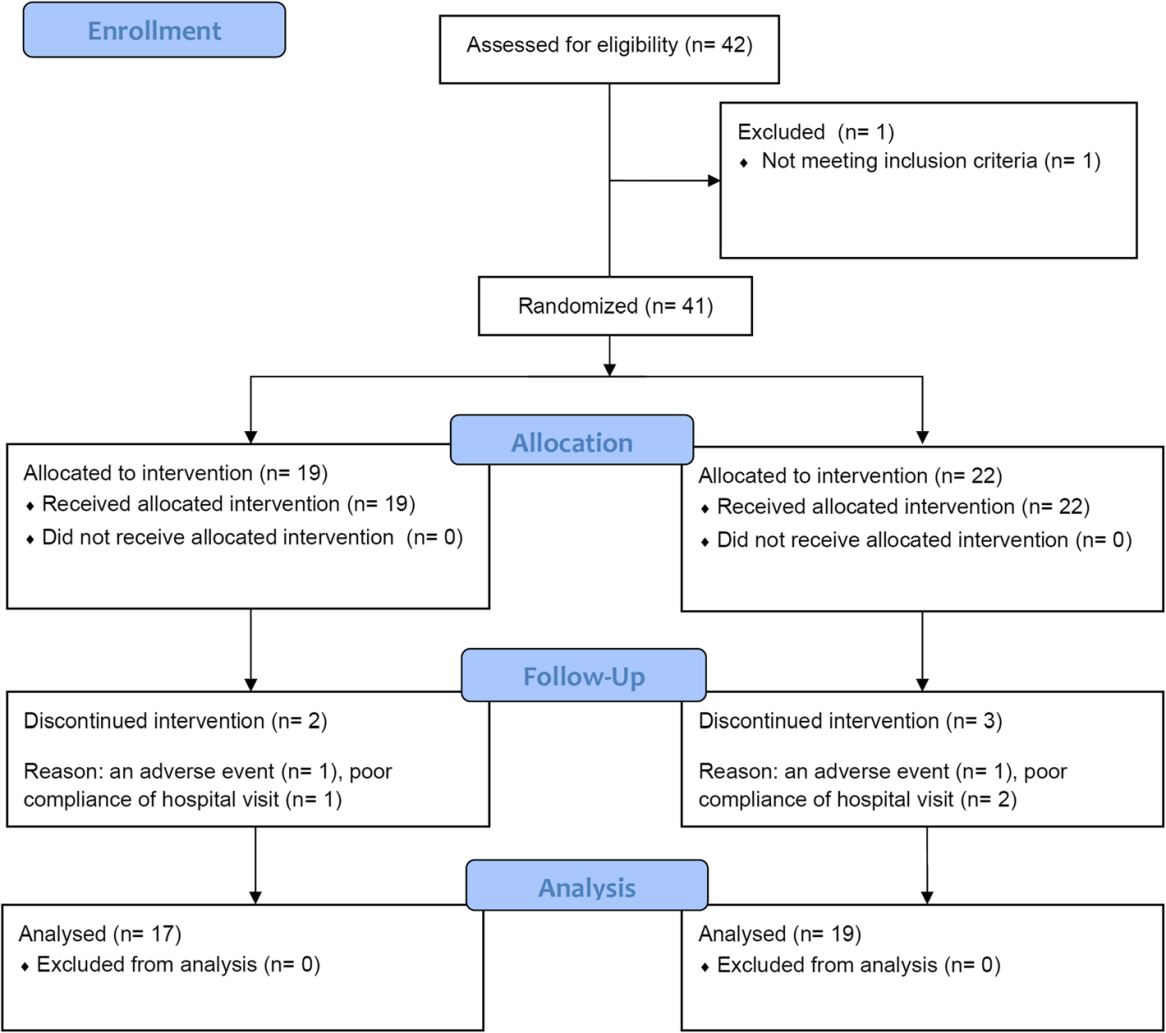
CONSORT flow of participants through the trial

Figure 1 shows the CONSORT diagram for the trial. Forty-one of 42 patients met the criteria and were enrolled in the study. When they were randomly allocated to the DCS-first (DCS was administered in the first phase) and PCB-first (PCB was administered in the first phase) groups, 19 and 22 patients were included in the groups, respectively. Seventeen (89.5%) and 19 (86.4%) patients completed the clinical study in the DCS-first and PCB-first groups, respectively, a total of 36 patients (Table 1). Five patients dropped out: 2 dropped out (1 each in the DCS-first and PCB-first groups) in the first treatment phase and 3 dropped out (1 and 2 in the DCS-first and PCB-first groups, respectively) in the second treatment phase. The reason for dropout was an adverse event (liver enzyme elevation) in 2 and poor compliance of hospital visit in 3. Both cases of adverse event occurred in the placebo phase.

**Table 1:** Patient characteristics

	DCS-first group	PCB-first group	Total
N (Male/Female)	17 (10/7)	19 (12/7)	36 (22/14)
Age (years)	48.4 ± 16.1	40.1 ± 10.7	44.0 ± 14.0
Age at onset (years)	22.6 ± 8.5	24.3 ± 5.6	23.5 ± 7.1
Age at starting therapy (years)	23.9 ± 8.5	24.8 ± 5.7	24.4 ± 7.1
Duration of illness (years)	25.8 ± 17.3	15.8 ± 9.2	20.5 ± 14.4
Education (years)	12.4 ± 2.6	13.9 ± 2.9	13.2 ± 2.9
CPZ equivalents (mg/day)	777.5 ± 614.8	871.3 ± 618.0	827.0 ± 609.5

*DCS* D-cycloserine, *PCB* placebo, *CPZ* chlorpromazine. No significant difference was noted between the DCS-first and PCB-first groups on age, age at onset, age at starting therapy, duration of illness, education, or CPZ equivalents

The male:female ratio of the subjects who completed the clinical study was 61%:39%, the mean age was 44 years old, the mean age at the time of treatment initiation was 24.4 years old, and the mean duration of illness was 20.5 years. The mean years of education was 13.2 years, and average chlorpromazine (CPZ) equivalent of antipsychotic drugs they took was 827 mg/day. No significant difference was noted between the DCS-first and PCB-first groups on age, age at onset, age at starting therapy, duration of illness, education, or CPZ equivalents. (Table 1).

The blood DCS levels were measured in each phase and compared by analysis of variance. There was no difference between baseline and PCB phase, and a significant difference between baseline and DCS phase, indicating that participants were adherent with the study protocol.

## Psychometrics

### Effect of treatment by time

No treatment by time effect was noted in any subscale of PANSS, SANS (Table 2), BACS, or EQS (Table 3). No treatment by time effect was noted in any of GAF, JCDSS, DIEPSS, and AIMS (data not shown).

**Table 2:** Treatment by time effect of DCS on PANSS and SANS

		Week within treatment phase								Analysis	
	Treatment	Baseline		Week 2		Week 4		Week 6		treatment x time	
		Mean	SD	Mean	SD	Mean	SD	Mean	SD	F	*p*
PANSS											
Positive	DCS	14.39	5.71	14.42	5.87	14.03	5.86	14.06	5.89	0.51	0.61
	PCB	14.72	5.79	14.19	5.46	14.11	5.16	13.97	5.62		
Negative	DCS	20.47	7.12	20.19	6.97	20.19	6.64	19.86	7.04	0.37	0.69
	PCB	20.58	6.87	20.31	7.01	20.00	6.57	19.56	7.11		
General	DCS	34.44	8.14	33.69	8.36	33.81	8.75	33.56	8.58	1.70	0.18
	PCB	35.22	8.11	34.56	7.78	33.97	7.26	33.83	8.48		
SANS											
I	DCS	13.97	8.39	13.56	8.65	13.67	8.51	13.36	8.40	0.34	0.79
	PCB	14.03	8.25	13.86	7.88	13.17	7.81	13.44	7.95		
II	DCS	7.92	4.92	7.39	5.06	7.31	4.83	7.11	4.89	0.44	0.72
	PCB	8.22	4.67	7.75	4.78	7.19	4.71	7.22	4.75		
III	DCS	10.14	4.26	9.89	4.45	9.78	4.32	9.61	4.32	0.50	0.62
	PCB	10.00	3.92	9.67	4.24	9.61	4.32	9.78	4.18		
IV	DCS	12.44	5.34	12.22	5.53	12.08	5.26	12.08	5.44	0.18	0.84
	PCB	12.50	5.01	12.33	5.35	12.14	5.16	12.47	5.36		
V	DCS	5.97	3.61	5.61	3.63	5.44	3.39	4.94	3.43	0.66	0.58
	PCB	5.78	3.69	5.64	3.77	4.78	3.39	5.03	3.34		
Summary	DCS	11.75	4.72	11.33	4.76	11.14	4.59	10.78	4.74	0.79	0.44
	PCB	11.42	4.76	11.39	4.56	10.83	4.58	10.89	4.59		
Total	DCS	50.44	21.88	48.67	22.57	48.28	21.69	47.11	22.55	0.52	0.62
	PCB	50.53	20.81	49.25	20.70	46.89	20.42	47.94	20.95		

*PANSS*, Positive and Negative Syndrome Scale; *SANS*, Scale for the Assessment of Negative Symptoms; *SD*, standard deviation; ‘Baseline’ indicates Week 0 for the first phase and Week 9 (after 3 week washout period) for the second phase

**Table 3:** Treatment by time effect of DCS on BACS and EQS

		Week within treatment phase				Analysis	
	Treatment	Baseline		Week 6		treatment x time	
		Mean	SD	Mean	SD	F	*p*
BACS							
VM	DCS	34.18	15.09	37.15	16.11	0.01	0.91
	PCB			37.15	16.04		
DS	DCS	16.48	5.76	16.97	5.96	0.20	0.65
	PCB			17.58	5.84		
TM	DCS	58.73	21.84	60.64	21.92	0.02	0.90
	PCB			63.33	20.69		
SC	DCS	46.52	16.36	47.03	15.71	0.89	0.35
	PCB			48.42	16.21		
VFC	DCS	15.21	4.70	16.64	6.34	4.06	0.053
	PCB			17.24	6.44		
VFL	DCS	17.55	9.58	18.12	8.23	1.06	0.31
	PCB			18.48	8.20		
TOL	DCS	14.73	5.57	15.70	5.62	0.13	0.72
	PCB			16.00	4.33		
EQS							
intra	DCS	39.29	14.40	41.04	18.23	0.08	0.78
	PCB			38.75	15.28		
inter	DCS	38.18	14.54	38.25	15.77	1.53	0.23
	PCB			38.75	15.28		
situa	DCS	30.79	16.89	33.46	18.66	0.00	0.99
	PCB			32.36	17.20		

*BACS* Brief Assessment of Cognition in Schizophrenia, *VM* verbal memory, *DS* digit sequencing task, *TM* token motor task, *SC* symbolic coding, *VFC* verbal fluency of category, *VFL* verbal fluency of letter, *TOL* tower of London, *EQS* Emotional Intelligence Scale, *intra* intrapersonal domain, *inter* interpersonal domain, situa, situational domain; ‘Baseline’ indicates Week 0 both for the first and second phase

## Differences of DCS-effect by onset age

**Fig. 2:**
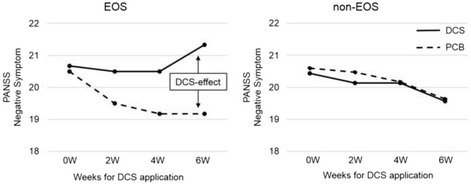
Changes in the scores of PANSS negative symptom PANSS, Positive and Negative Syndrome Scale; DCS-effect was defined by scores at DCS phase subtracted by those at PCB phase. In the non-EOS group, the scores for both DCS and PCB declined as time proceeded and almost no difference (−0.07) was noted in the DCS-effect at 6 W. In the EOS group, the score for PCB declined as time proceeded whereas the one for DCS demonstrated less decline or even an increase, resulting in the positive value (+2.2) for the DCS-effect at 6 W

There was no overall effect of onset age on PANSS subscales (F(3, 31) = 2.20, *p* = 0.11). However, there was a significant effect of onset age on negative symptoms (F(1, 33) = 6.17, *p* = 0.02) (Table 4). In the non-EOS group, the scores for both DCS and PCB declined as time proceeded and almost no difference was noted in the DCS-effect at 6 W (the score of DCS phase subtracted by the one of PCB phase was −0.07). In the EOS group, the score for PCB declined as time proceeded whereas the one for DCS demonstrated less decline or even an increase, resulting in the positive value for the DCS-effect at 6 W (the score of DCS phase subtracted by the one of PCB phase was +2.2) (Fig. 2). No significant difference was noted in the overall effect between EOS and non-EOS on SANS, BACS, or EQS, and no significant difference was noted in any subscale. None of the psychometrics ratings of one patient with late onset were outliers.

**Table 4:** Differences of DCS-effect by onset age

		DCS-effect	SD	F value	*p* value
PANSS				2.20	0.11
Positive	EOS	0.83	1.83	1.54	0.22
	non-EOS	−0.07	1.39		
Negative	EOS	2.17	2.79	6.17	**0.02**
	non-EOS	−0.07	1.76		
General	EOS	1.33	3.50	1.72	0.20
	non-EOS	−0.60	3.07		
SANS				1.11	0.38
I	EOS	1.50	2.74	2.02	0.16
	non-EOS	−0.40	2.49		
II	EOS	0.50	0.84	0.57	0.46
	non-EOS	−0.23	1.72		
III	EOS	−0.50	1.52	0.20	0.66
	non-EOS	−0.10	1.60		
IV	EOS	0.17	1.33	0.54	0.47
	non-EOS	−0.50	1.81		
V	EOS	0.67	1.51	0.70	0.41
	non-EOS	−0.23	1.76		
Summary	EOS	0.83	1.83	1.95	0.17
	non-EOS	−0.30	1.51		
Total	EOS	2.33	5.82	0.92	0.34
	non-EOS	−1.47	7.22		
BACS				0.85	0.56
VM	EOS	−2.83	9.28	1.64	0.21
	non-EOS	0.68	5.12		
DS	EOS	−0.17	2.32	0.19	0.66
	non-EOS	−0.68	3.42		
TM	EOS	0.67	5.75	0.47	0.50
	non-EOS	−3.96	14.86		
SC	EOS	−1.67	6.12	0.01	0.94
	non-EOS	−1.39	7.02		
VFC	EOS	−3.17	3.54	2.69	0.11
	non-EOS	0.04	3.70		
VFL	EOS	0.67	3.14	0.51	0.48
	non-EOS	−0.61	5.79		
TOL	EOS	0.00	3.69	0.02	0.90
	non-EOS	−0.29	3.75		
EQS				1.18	0.34
intra	EOS	−0.60	12.82	0.86	0.36
	non-EOS	2.58	6.10		
inter	EOS	−2.40	9.21	0.65	0.43
	non-EOS	0.17	8.69		
situa	EOS	3.00	6.12	0.53	0.47
	non-EOS	0.79	6.06		

*PANSS* Positive and Negative Syndrome Scale, *SANS* Scale for the Assessment of Negative Symptoms, *BACS* Brief Assessment of Cognition in Schizophrenia, *VM* verbal memory, *DS* digit sequencing task, *TM* token motor task, *SC* symbolic coding, *VFC* verbal fluency of category, *VFL* verbal fluency of letter, *TOL* tower of London, *EQS* Emotional Intelligence Scale, *intra* intrapersonal domain, *inter* interpersonal domain, *situa* situational domain, *SD* standard deviation; DCS-effect, scores at DCS phase extracted by those at PCB phase

## MR diffusion tensor imaging

Data could be collected from 21 of the 36 patients who completed the study. These participants were those who agreed and traveled to TMDU for the MRI scan, comprising the 3 EOS and 18 non-EOS patients. Regarding the subscales of PANSS, SANS, BACS, and EQS, the correlation between the FA value of each brain region and DCS-effect was investigated (Table 5). The significant correlations (*p* < 0.01) were demonstrated in PANSS-G, SANS-IV, BACS-TM, and BACS-TOL.

**Table 5:** Correlation between the FA value of each brain region and DCS-effect

		PANSS			SANS							BACS							EQS		
		P	N	G	I	II	III	IV	V	Sumarry	Total	VM	DS	TM	SC	VFC	FVL	TOL	intra	inter	situa
middle cerebellar peduncle	r	−.014	−.242	−.023	.071	.139	−.120	−.061	−.304	−.310	−.104	.070	.344	.281	.042	−.243	−.034	.012	.081	−.414	−.375
	p	.952	.290	.922	.759	.549	.605	.793	.180	.171	.654	.769	.137	.230	.859	.302	.888	.960	.782	.141	.187
	n	21	21	21	21	21	21	21	21	21	21	20	20	20	20	20	20	20	14	14	14
pontine crossing tract	r	.291	**−.539** ^*^	−.140	−.187	.173	.051	−.373	−.028	−.302	−.134	−.070	.365	.392	−.080	.079	.171	.180	.162	−.107	−.269
	p	.201	**.012**	.545	.417	.453	.827	.096	.906	.184	.563	.768	.114	.087	.738	.740	.472	.447	.581	.716	.352
	n	21	21	21	21	21	21	21	21	21	21	20	20	20	20	20	20	20	14	14	14
genu of corpus callosum	r	.189	.121	.303	−.078	.148	.292	.123	.095	.072	.169	−.443	−.076	.228	−.095	−.214	−.007	**.585** ^**^	.066	.380	.253
	p	.412	.602	.182	.735	.522	.200	.596	.682	.756	.464	.050	.751	.334	.689	.365	.977	**.007**	.822	.181	.383
	n	21	21	21	21	21	21	21	21	21	21	20	20	20	20	20	20	20	14	14	14
body of corpus callosum	r	.386	−.082	−.042	−.013	.358	.156	−.110	.170	.087	.161	−.102	.182	.283	.140	−.230	−.011	**.460** ^*^	−.073	.429	.108
	p	.084	.723	.858	.957	.111	.499	.636	.462	.708	.485	.670	.442	.226	.557	.329	.962	**.041**	.804	.126	.714
	n	21	21	21	21	21	21	21	21	21	21	20	20	20	20	20	20	20	14	14	14
splenium of corpus callosum	r	**−.444** ^*^	−.432	**−.574** ^**^	**−.439** ^*^	−.115	.212	−.404	−.353	−.347	−.378	.092	.318	−.235	−.015	.121	−.136	−.202	−.325	−.182	−.163
	p	**.044**	.050	**.007**	**.047**	.621	.356	.069	.117	.123	.091	.699	.171	.318	.949	.611	.568	.393	.257	.534	.577
	n	21	21	21	21	21	21	21	21	21	21	20	20	20	20	20	20	20	14	14	14
fornix	r	.334	.052	.146	−.089	.319	.180	−.120	.082	.082	.096	−.095	.200	**.517** ^*^	−.067	.017	−.062	**.519** ^*^	.090	.471	.412
	p	.139	.823	.529	.703	.158	.435	.605	.725	.725	.680	.689	.397	**.020**	.780	.942	.797	**.019**	.759	.089	.143
	n	21	21	21	21	21	21	21	21	21	21	20	20	20	20	20	20	20	14	14	14
corticospinal tract R	r	.300	−.120	.023	−.096	.066	−.167	.063	−.132	−.141	−.099	.304	.196	.021	.197	.189	**.530** ^*^	−.127	.173	−.431	−.038
	p	.187	.605	.923	.678	.775	.468	.787	.567	.543	.669	.193	.408	.928	.406	.425	**.016**	.593	.555	.124	.898
	n	21	21	21	21	21	21	21	21	21	21	20	20	20	20	20	20	20	14	14	14
corticospinal tract L	r	.129	−.023	−.013	−.055	.107	−.313	.085	−.159	−.084	−.121	.435	.200	−.045	.258	.219	**.471** ^*^	−.227	.161	−.278	−.053
	p	.576	.920	.955	.811	.645	.167	.713	.492	.716	.601	.056	.397	.851	.272	.353	**.036**	.336	.582	.335	.858
	n	21	21	21	21	21	21	21	21	21	21	20	20	20	20	20	20	20	14	14	14
medial lemniscus R	r	.255	.193	−.011	−.120	.136	.193	.188	−.114	−.022	.064	.141	−.082	.249	.232	−.034	.118	.342	−.287	−.058	.131
	p	.265	.402	.961	.605	.557	.403	.414	.622	.925	.782	.553	.732	.289	.326	.885	.621	.141	.319	.844	.654
	n	21	21	21	21	21	21	21	21	21	21	20	20	20	20	20	20	20	14	14	14
medial lemniscus L	r	.236	.023	−.188	−.206	.113	.066	.067	−.154	−.078	−.064	.189	.094	.219	.287	.031	.163	.308	−.270	.058	.104
	p	.303	.920	.415	.369	.625	.777	.774	.505	.736	.784	.425	.693	.354	.220	.896	.493	.186	.350	.845	.723
	n	21	21	21	21	21	21	21	21	21	21	20	20	20	20	20	20	20	14	14	14
inferior cerebellar peduncle R	r	.367	−.094	−.181	.020	−.065	.324	−.040	−.043	−.082	.063	−.179	.040	.187	−.277	−.111	.131	.397	−.241	−.105	.008
	p	.102	.685	.433	.932	.780	.152	.863	.854	.723	.787	.451	.868	.430	.238	.642	.583	.083	.406	.721	.977
	n	21	21	21	21	21	21	21	21	21	21	20	20	20	20	20	20	20	14	14	14
inferior cerebellar peduncle L	r	.396	−.133	−.212	.025	.121	.268	−.061	.118	.017	.146	−.105	.062	.252	−.078	.022	.268	.354	−.146	.126	.018
	p	.076	.566	.357	.915	.601	.240	.791	.610	.942	.528	.660	.796	.284	.745	.926	.254	.126	.618	.667	.952
	n	21	21	21	21	21	21	21	21	21	21	20	20	20	20	20	20	20	14	14	14
superior cerebellar peduncle R	r	−.203	−.091	−.349	−.012	−.234	−.186	−.212	−.233	−.225	−.269	.296	.217	.015	.330	.083	.179	.105	−.156	−.071	−.152
	p	.376	.694	.121	.959	.307	.420	.357	.310	.327	.238	.204	.359	.949	.156	.729	.449	.660	.594	.808	.605
	n	21	21	21	21	21	21	21	21	21	21	20	20	20	20	20	20	20	14	14	14
superior cerebellar peduncle L	r	−.186	−.229	**−.484** ^*^	−.006	−.361	−.247	−.263	−.313	−.379	−.362	.153	.245	.118	.104	.084	.174	.166	−.253	−.153	−.161
	p	.419	.319	**.026**	.978	.108	.280	.249	.167	.090	.107	.519	.298	.620	.662	.726	.462	.483	.384	.601	.582
	n	21	21	21	21	21	21	21	21	21	21	20	20	20	20	20	20	20	14	14	14
cereberal peduncle R	r	.075	−.164	−.245	.125	−.055	.028	−.221	−.085	−.169	−.057	−.089	.178	**.465** ^*^	.196	.082	−.050	.049	.023	.043	.479
	p	.748	.477	.285	.590	.812	.905	.336	.716	.465	.807	.708	.452	**.039**	.406	.731	.833	.838	.938	.885	.083
	n	21	21	21	21	21	21	21	21	21	21	20	20	20	20	20	20	20	14	14	14
cereberal peduncle L	r	−.307	−.053	−.179	−.003	−.180	.260	−.189	−.145	−.131	−.077	−.358	.074	−.013	−.048	−.016	−.129	.324	−.022	.147	.257
	p	.175	.818	.438	.989	.435	.255	.413	.532	.572	.741	.121	.758	.956	.842	.946	.587	.164	.941	.617	.375
	n	21	21	21	21	21	21	21	21	21	21	20	20	20	20	20	20	20	14	14	14
anterior limb of internal capsule R	r	.189	.220	.315	.101	.054	.353	.202	.028	.157	.231	.015	−.225	.295	.325	.371	−.163	.223	−.275	.085	.354
	p	.412	.339	.165	.663	.815	.116	.380	.903	.497	.314	.950	.341	.207	.162	.107	.493	.344	.342	.772	.214
	n	21	21	21	21	21	21	21	21	21	21	20	20	20	20	20	20	20	14	14	14
anterior limb of internal capsule L	r	.033	.086	.262	−.136	−.143	.310	.151	.002	−.057	.055	−.425	−.239	.083	.050	.014	−.195	**.463** ^*^	−.118	.070	.216
	p	.886	.710	.252	.558	.537	.171	.514	.993	.807	.813	.062	.311	.728	.834	.952	.411	**.040**	.689	.813	.459
	n	21	21	21	21	21	21	21	21	21	21	20	20	20	20	20	20	20	14	14	14
posterior limb of internal capsule R	r	−.413	−.326	−.369	−.323	−.113	.144	−.402	−.395	−.303	−.370	−.018	.198	−.003	.258	.356	−.218	−.422	.015	−.087	.287
	p	.063	.149	.100	.154	.624	.533	.071	.076	.181	.099	.940	.403	.989	.272	.123	.356	.064	.960	.767	.320
	n	21	21	21	21	21	21	21	21	21	21	20	20	20	20	20	20	20	14	14	14
posterior limb of internal capsule L	r	−.383	−.363	−.251	−.288	−.180	.153	−.177	−.394	−.239	−.302	.083	.235	**−.477** ^*^	.112	.193	−.098	−.433	−.223	−.216	−.141
	p	.087	.106	.272	.205	.436	.507	.444	.077	.297	.183	.728	.318	**.033**	.639	.416	.682	.057	.444	.459	.630
	n	21	21	21	21	21	21	21	21	21	21	20	20	20	20	20	20	20	14	14	14
retrolenticular part of internal capsule R	r	**−.465** ^*^	.224	−.008	−.293	−.353	−.118	.064	−.377	−.270	−.352	−.153	−.380	−.105	.255	−.033	−.307	−.256	.079	−.118	**.604** ^*^
	p	**.034**	.330	.972	.197	.116	.609	.783	.092	.236	.118	.519	.098	.660	.277	.889	.188	.277	.789	.687	**.022**
	n	21	21	21	21	21	21	21	21	21	21	20	20	20	20	20	20	20	14	14	14
retrolenticular part of internal capsule L	r	−.249	−.021	.030	−.017	.052	.167	.044	−.202	−.090	−.002	−.057	−.056	−.072	.270	−.039	.105	−.319	.418	−.095	.329
	p	.276	.927	.898	.942	.824	.469	.850	.380	.698	.993	.811	.816	.763	.249	.871	.659	.171	.137	.746	.251
	n	21	21	21	21	21	21	21	21	21	21	20	20	20	20	20	20	20	14	14	14
anterior corona radiata R	r	.246	.285	**.440** ^*^	−.248	.045	.213	.386	−.088	−.036	.070	.082	−.189	.170	−.058	.114	−.181	.353	−.256	−.156	.218
	p	.282	.210	**.046**	.279	.846	.354	.084	.704	.876	.764	.731	.424	.474	.807	.631	.444	.126	.378	.594	.454
	n	21	21	21	21	21	21	21	21	21	21	20	20	20	20	20	20	20	14	14	14
anterior corona radiata L	r	.052	.081	.197	−.421	.152	.257	.204	−.120	−.079	−.021	−.003	−.012	.186	−.207	.157	−.176	.129	−.117	−.045	.286
	p	.824	.727	.391	.057	.510	.260	.375	.605	.734	.928	.991	.959	.432	.381	.508	.457	.586	.690	.879	.322
	n	21	21	21	21	21	21	21	21	21	21	20	20	20	20	20	20	20	14	14	14
superior corona radiata R	r	−.272	.141	−.059	.148	.045	.364	.053	−.049	.144	.176	.112	−.141	−.264	.243	.051	−.290	.042	−.385	−.044	−.075
	p	.233	.541	.798	.522	.846	.105	.820	.832	.532	.445	.640	.554	.262	.302	.831	.215	.861	.174	.880	.799
	n	21	21	21	21	21	21	21	21	21	21	20	20	20	20	20	20	20	14	14	14
superior corona radiata L	r	−.100	.196	.007	−.026	.102	.398	.295	.055	.290	.247	.181	−.170	−.401	.316	−.027	−.066	−.021	**−.614** ^*^	−.011	−.133
	p	.668	.395	.974	.910	.661	.074	.194	.812	.202	.280	.445	.473	.080	.175	.912	.782	.932	**.019**	.971	.650
	n	21	21	21	21	21	21	21	21	21	21	20	20	20	20	20	20	20	14	14	14
posterior corona radiata R	r	−.374	−.058	−.111	−.073	.178	.169	.067	−.137	.054	.040	−.241	−.186	−.292	.110	−.166	−.244	**−.529** ^*^	−.120	−.195	−.036
	p	.095	.804	.633	.754	.439	.463	.772	.552	.816	.862	.306	.433	.211	.645	.485	.300	**.017**	.683	.505	.904
	n	21	21	21	21	21	21	21	21	21	21	20	20	20	20	20	20	**20**	14	14	14
posterior corona radiata L	r	−.154	.013	.021	−.006	.136	.178	.346	−.043	.174	.176	−.198	−.228	−.357	.043	−.190	−.099	−.312	**−.549** ^*^	−.274	−.481
	p	.505	.955	.927	.980	.557	.440	.125	.853	.451	.445	.402	.333	.123	.858	.422	.678	.180	**.042**	.343	.082
	n	21	21	21	21	21	21	21	21	21	21	20	20	20	20	20	20	20	14	14	14
posterior thalamic radiation R	r	.238	.026	.223	−.341	.079	−.013	.198	−.265	−.259	−.147	.019	.010	.268	.137	−.202	−.118	−.135	.150	−.315	**.553** ^*^
	p	.298	.910	.331	.130	.733	.955	.389	.245	.257	.525	.936	.965	.253	.565	.393	.619	.570	.609	.272	**.040**
	n	21	21	21	21	21	21	21	21	21	21	20	20	20	20	20	20	20	14	14	14
posterior thalamic radiation L	r	.183	**.443** ^*^	**.571** ^**^	−.010	−.005	.147	**.598** ^**^	−.008	.111	.219	−.195	−.389	.016	.107	−.352	−.113	.275	.039	−.094	.353
	p	.427	**.044**	**.007**	.965	.983	.526	**.004**	.972	.633	.340	.409	.090	.946	.653	.128	.634	.241	.895	.748	.216
	n	21	21	21	21	21	21	21	21	21	21	20	20	20	20	20	20	20	14	14	14
sagittal stratum R	r	.190	−.045	.224	−.240	.262	−.029	.186	−.014	−.106	.019	−.025	.085	**.521** ^*^	.254	.010	.030	−.048	.369	.131	**.615** ^*^
	p	.410	.848	.328	.295	.251	.899	.419	.953	.647	.934	.916	.721	**.019**	.280	.967	.900	.840	.194	.656	**.019**
	n	21	21	21	21	21	21	21	21	21	21	20	20	20	20	20	20	20	14	14	14
sagittal stratum L	r	.191	.080	.170	−.301	.318	−.118	.224	−.015	−.078	−.005	.064	.178	**.596** ^**^	.061	−.088	−.140	.285	.110	.231	.355
	p	.406	.731	.462	.185	.160	.610	.329	.947	.736	.981	.789	.453	**.006**	.799	.713	.555	.224	.708	.428	.212
	n	21	21	21	21	21	21	21	21	21	21	20	20	20	20	20	20	20	14	14	14
external capsule R	r	.300	.167	.059	−.027	.172	−.030	.076	.097	−.112	.082	−.032	−.182	**.515** ^*^	.253	−.217	−.049	**.559** ^*^	−.019	.179	.461
	p	.186	.469	.800	.908	.455	.896	.743	.677	.629	.723	.894	.442	**.020**	.283	.357	.836	**.010**	.949	.541	.097
	n	21	21	21	21	21	21	21	21	21	21	20	20	20	20	20	20	20	14	14	14
external capsule L	r	.179	.166	.083	.095	.172	.023	.041	.229	−.020	.181	−.235	−.184	.386	.002	−.205	−.180	**.702** ^**^	−.051	.291	.228
	p	.437	.471	.721	.681	.456	.923	.860	.319	.932	.432	.319	.437	.092	.994	.386	.448	**.001**	.862	.313	.432
	n	21	21	21	21	21	21	21	21	21	21	20	20	20	20	20	20	20	14	14	14
cingulum (cingulate gyrus) R	r	**.455** ^*^	.164	.080	.116	−.028	−.032	.227	.195	−.023	.166	−.098	−.029	**.584** ^**^	.078	−.111	.169	**.869** ^**^	−.041	.211	.221
	p	**.038**	.477	.732	.618	.904	.891	.322	.396	.922	.472	.680	.903	**.007**	.743	.641	.476	**.000**	.890	.470	.448
	n	21	21	21	21	21	21	21	21	21	21	20	20	20	20	20	20	20	14	14	14
cingulum (cingulate gyrus) L	r	**.481** ^*^	.126	.307	.169	−.098	−.037	.323	.099	−.148	.161	−.259	−.216	**.575** ^**^	.003	−.159	−.159	**.664** ^**^	−.152	−.126	.185
	p	**.027**	.587	.176	.463	.672	.873	.153	.669	.523	.486	.270	.361	**.008**	.989	.503	.503	**.001**	.605	.669	.527
	n	21	21	21	21	21	21	21	21	21	21	20	20	20	20	20	20	**20**	14	14	14
cingulum (hippocampus) R	r	.196	.095	.036	−.029	−.202	−.203	.041	−.204	−.268	−.185	−.166	−.087	**.543** ^*^	.084	.048	−.084	.370	−.068	−.001	.382
	p	.395	.682	.878	.900	.380	.378	.862	.376	.240	.422	.483	.715	**.013**	.724	.839	.724	.109	.816	.998	.178
	n	21	21	21	21	21	21	21	21	21	21	20	20	20	20	20	20	20	14	14	14
cingulum (hippocampus) L	r	.187	−.183	−.229	.141	−.064	−.220	−.104	−.050	−.130	−.080	−.150	.100	.291	.141	.061	.046	.403	−.215	.170	−.035
	p	.417	.426	.318	.541	.784	.339	.655	.830	.574	.730	.527	.676	.214	.553	.797	.848	.078	.461	.562	.905
	n	21	21	21	21	21	21	21	21	21	21	20	20	20	20	20	20	20	14	14	14
fornix stria terminalis R	r	**.451** ^*^	−.032	.097	.031	−.061	−.043	−.115	.070	−.252	−.027	−.059	−.056	**.668** ^**^	.014	.088	.013	.357	.219	−.132	**.561** ^*^
	p	**.040**	.892	.675	.893	.794	.852	.618	.763	.271	.909	.805	.813	**.001**	.955	.711	.956	.122	.451	.654	**.037**
	n	21	21	21	21	21	21	21	21	21	21	20	20	20	20	20	20	20	14	14	14
fornix stria terminalis L	r	.267	.103	.084	.104	.127	.371	−.198	.159	−.006	.182	−.312	−.136	**.472** ^*^	−.147	−.119	−.138	**.581** ^**^	.206	.171	.429
	p	.243	.656	.716	.652	.583	.098	.391	.491	.980	.430	.180	.569	**.036**	.537	.618	.563	**.007**	.481	.559	.126
	n	21	21	21	21	21	21	21	21	21	21	20	20	20	20	20	20	20	14	14	14
superior longitudinal fasciculus R	r	−.025	.033	−.121	−.270	.052	.310	−.173	−.117	−.151	−.087	−.061	.074	.361	.200	.105	.201	**.483** ^*^	.061	.116	.420
	p	.913	.888	.603	.237	.821	.172	.453	.614	.514	.707	.798	.757	.118	.397	.658	.396	**.031**	.835	.692	.135
	n	21	21	21	21	21	21	21	21	21	21	20	20	20	20	20	20	20	14	14	14
superior longitudinal fasciculus L	r	−.262	.218	−.103	−.147	.228	.260	.123	−.033	.233	.107	.099	−.007	.020	.311	.025	.295	.091	.132	.412	.287
	p	.252	.343	.658	.524	.320	.256	.595	.887	.310	.643	.679	.978	.933	.183	.917	.207	.703	.652	.143	.320
	n	21	21	21	21	21	21	21	21	21	21	20	20	20	20	20	20	20	14	14	14
superior front occipital fasciculus R	r	−.260	.213	.212	.003	−.117	.328	.135	−.163	−.016	.054	−.136	−.261	−.095	.126	−.144	−.435	−.036	−.248	−.156	.370
	p	.255	.354	.357	.991	.613	.147	.559	.479	.945	.817	.567	.266	.689	.596	.546	.056	.881	.392	.595	.192
	n	21	21	21	21	21	21	21	21	21	21	20	20	20	20	20	20	20	14	14	14
superior front occipital fasciculus L	r	**−.484** ^*^	−.185	−.129	−.116	−.135	.398	−.085	−.114	−.009	−.023	−.220	−.040	−.397	.145	.188	−.051	**−.494** ^*^	.050	−.053	.196
	p	**.026**	.422	.577	.617	.558	.074	.715	.622	.970	.922	.350	.868	.083	.541	.428	.831	**.027**	.866	.858	.503
	n	21	21	21	21	21	21	21	21	21	21	20	20	20	20	20	20	20	14	14	14
uncinate fasciculus R	r	.286	.159	.047	.004	.133	−.072	−.054	−.038	−.044	−.017	.122	.020	**.489** ^*^	.366	−.045	.104	**.485** ^*^	.064	.372	.496
	p	.210	.490	.838	.987	.565	.756	.818	.871	.848	.941	.608	.934	**.029**	.113	.849	.662	**.030**	.827	.191	.071
	n	21	21	21	21	21	21	21	21	21	21	20	20	20	20	20	20	20	14	14	14
uncinate fasciculus L	r	.187	.271	.170	.167	.096	.027	.157	.153	.048	.198	.045	−.263	.219	.223	−.136	−.049	**.484** ^*^	−.032	.291	.298
	p	.416	.234	.462	.470	.678	.908	.496	.508	.836	.390	.850	.263	.355	.344	.568	.837	**.031**	.913	.313	.300
	n	21	21	21	21	21	21	21	21	21	21	20	20	20	20	20	20	20	14	14	14
tapetum R	r	.128	−.075	−.095	−.280	.302	.288	−.088	−.211	−.117	−.043	.046	.021	.185	.161	−.196	−.144	−.155	−.066	−.242	.176
	p	.580	.745	.683	.219	.183	.206	.705	.359	.612	.853	.848	.930	.436	.498	.408	.545	.514	.822	.404	.548
	n	21	21	21	21	21	21	21	21	21	21	20	20	20	20	20	20	20	14	14	14
tapetum L	r	.077	.106	.021	.151	.054	.426	.018	−.023	.029	.197	.107	−.172	−.100	−.130	−.339	−.367	.117	−.495	−.294	−.259
	p	.740	.648	.928	.515	.817	.054	.940	.920	.902	.392	.652	.468	.674	.584	.144	.111	.624	.072	.307	.370
	n	21	21	21	21	21	21	21	21	21	21	20	20	20	20	20	20	20	14	14	14

r, Pearson’s correlation coefficient; *. *p* < 0.05 **. *p* < 0.01; *R* right, *L* left, *PANSS* Positive and Negative Syndrome Scale, *SANS* Scale for the Assessment of Negative Symptoms, *BACS* Brief Assessment of Cognition in Schizophrenia, *VM* verbal memory, *DS* digit sequencing task, *TM* token motor task, *SC* symbolic coding, *VFC* verbal fluency of category, *VFL* verbal fluency of letter, *TOL* tower of London, *EQS* Emotional Intelligence Scale, *intra* intrapersonal domain, *inter* interpersonal domain, *situa* situational domain; Data in bold indicate they are significant values

In PANSS-G, an inverse correlation with FA of the splenium of the corpus callosum was noted (the more grade of improvement by DCS was associated with higher FA), and a positive correlation with FA of the posterior thalamic radiation left (L) was noted (the more grade of improvement was associated with lower FA).

In SANS-IV, a positive correlation with FA of the posterior thalamic radiation L was noted (the more grade of improvement was associated with lower FA).

In BACS-TM, positive correlations with FA of the following 4 regions were noted: the sagittal stratum L, cingulum (cingulate gyrus) right (R), cingulum (cingulate gyrus) L, and fornix stria terminalis R (the more grade of improvement was associated with higher FA).

In BACS-TOL, positive correlations with FA of the following 5 regions were noted: the genu of corpus callosum, external capsule L, cingulum (cingulate gyrus) R, cingulum (cingulate gyrus) L, and fornix stria terminalis L (the more grade of improvement was associated with higher FA).

Of these significant correlations, if the 3 EOS patients were excluded from the analysis, the correlations between BACS-TM and cingulum (cingulate gyrus) R and BACS-TOL and fornix stria terminalis L turned non-significant in the *p* < 0.01 criteria (*p* = 0.011 and 0.016, respectively).

## Discussion

The main finding of this study was that DCS did not improve positive symptoms, negative symptoms, or cognitive dysfunction of schizophrenia, and these findings were consistent with most preceding studies concluding that DCS is ineffective for positive symptoms. Regarding negative symptoms, the findings were inconsistent with some preceding studies in which improvement by DCS was observed [6, 9, 11, 14–16], but consistent with other preceding studies in which no improvement by DCS was observed [10, 13, 18–20] or the condition aggravated [7, 8]. Fewer studies evaluated cognitive dysfunction, but the condition was unchanged [9–11, 19, 20] excluding a study reporting an improvement [6], being consistent with our study. In one meta-analysis including 7 double-blind placebo controlled studies [27], DCS did not improve any domain of symptoms, being consistent with our study. However, we can’t exclude the possibility that our study failed to demonstrate significant main effects due to the lack of statistical power.

It is possible that the absence of the effect of DCS in this study may have been due to the narrow therapeutic window of DCS. In a dose finding study in which DCS was administered to 8 schizophrenia patients at 15, 50, and 250 mg, negative symptoms were most markedly improved at 50 mg [6], whereas symptoms aggravated when DCS was administered at 100 mg or higher [13, 17]. It was assumed that since DCS is a partial agonist, it competes with endogenous glycine for the NMDA receptor glycine binding site acting as an antagonist. This was the initial study involving individuals of Japanese descent, and no Japanese individual may have been included in preceding studies including the dose finding study. It is possible that DCS 50 mg is not an optimum dose for Japanese and it may not have changed symptoms in this study.

DCS was administered in addition to antipsychotic drugs that the patients usually took. In preceding clinical studies, when DCS was administered to patients usually treated with clozapine, negative symptoms aggravated [7, 8]. It has also been reported that the grade of improvement of the negative symptom score was greater in patients usually treated with typical than atypical antipsychotic drugs [16]. In the current study, no subject was medicated with clozapine but most subjects were medicated with atypical antipsychotic drugs, suggesting that DCS did not exhibit a significant effect on negative symptoms in appearance.

DCS was administered at 50 mg/day daily for 6 weeks, but no improvement of cognitive dysfunction was noted. According to Goff et al. [11], DCS administered at 50 mg weekly for 8 weeks did not improve overall cognitive function, but improvement was observed in a memory reconstruction task 7 days after a single administration of DCS. Regarding the cognitive function, repeated daily administration of DCS may cause desensitization of the NMDA receptor. In animal model, desensitization was observed with repeated daily administration of DCS [28, 29]. Another evidence suggest that elevated co-agonists of glycine recognition site facilitate the internalization of NMDA receptor from the cell surface [30], representing its desensitization. Since DCS was administered daily repeatedly in our study, it may have caused the desensitization resulting in the absence of the effect of DCS on cognitive dysfunction.

The influence of onset age on the DCS-effect was investigated in the secondary analysis, in which the score was mostly aggravated in the EOS group compared with that in the non-EOS group (i.e., the PANSS and SANS scores increased and the BACS and EQS scores decreased) (Table 4), and aggravation of the PANSS score of negative symptoms in the EOS group was significantly different from that in the non-EOS group. Generally, early-onset schizophrenia (EOS) is intractable and the prognosis is poor compared with non-early-onset schizophrenia (non-EOS). In addition, in our previous clinical genetic study in consideration of the onset age, gene expression related to glutamate neurotransmission was different between EOS and non-EOS [31]. Findings observed in these and our studies suggested that heterogeneity of the system related to glutamate neurotransmission appears as a differences in the onset age of schizophrenia and influences response to treatment. However, the findings of the current study might be statistically limited due to the small number of EOS (*n* = 6) and unbalanced distribution between EOS and non-EOS (*n* = 30).

By MR-DTI, evidence of altered FA in brain regions of the focus of our study has been accumulated, i.e., decreased FA in genu and splenium of corpus callosum has been reported in patients with schizophrenia [32]. Similarly, bilaterally reduced FA was demonstrated in both anterior and posterior cingulum bundles, and suggested associations between FA and the symptomatology of schizophrenia [33, 34]. Altered FA was reported in brain regions including sagittal stratum and external capsule in suicide attempters with schizophrenia [35]. Reduced FA and increased MD were found in the fornix of patients with schizophrenia, and increased MD was associated with performance in cognitive tasks [36]. Although no physiological or pathophysiological significance of FA values has been established, it is assumed to be related to changes in the density and damage of the white matter. In the current study, a symptom-site-specific correlation was noted between the treatment effect of DCS and FA. The better treatment effect of DCS on BACS was observed when FA was higher in the sagittal stratum, cingulum, fornix stria terminalis, genu of corpus callosum, and external capsule, and the better treatment effect on PANSS-G was observed when FA of the splenium of corpus callosum was higher. In contrast, the better treatment effect of DCS on PANSS-G and SANS-IV was observed when FA was lower in the posterior thalamic radiation (left). To our knowledge, there is no study that has investigated associations between treatment effect of DCS and white matter integrity. A study reporting that clozapine treatment increased FA in various brain regions in patients with schizophrenia is suggestive to the findings of our study [37]. Although our study did not examine changes of FA by DCS treatment per se, changes in cells or molecules in the white matter in the specific brain regions or the grade of subsequent abnormalities of anatomical and functional connectivity between specific brain regions may influence the treatment effect of DCS. Possible aspects of interpreting our findings include that: (1) DCS improves the cognitive function and PANSS-G when the tract is retained at a specific level in brain regions other than the posterior thalamic radiation described above, (2) changes in connectivity with a part of the cortex closely connected with the posterior thalamic radiation through fibers is related the DCS action, and (3) DCS treatment possibly improved the FA of, e.g. thalamic radiation, where an asymmetric alteration in the white matter integrity was demonstrated [38].

## Conclusion

In conclusion, DCS did not improve positive symptoms, negative symptoms, or cognitive dysfunction in schizophrenia. It was suggested that response to DCS is influenced by heterogeneity derived from differences in the onset age and white matter integrity.

## Abbreviations

AIMS: Abnormal Involuntary Movement Scale
BACS: Brief Assessment of Cognition in Schizophrenia
CPZ: chlorpromazine
CREST: Core Research for Evolutional Science and Technology
DCS: D-cycloserine
DIEPSS: Drug Induced Extra-Pyramidal Symptoms Scale
DS: digit sequencing task
DSM: Diagnostic and Statistical Manual of Mental Disorders
DTI: diffusion tensor imaging
EOS: early-onset
EQS: Emotional Intelligence Scale
FA: fractional anisotropy
FA, MD, AD, RD maps: fractional anisotropy, mean diffusivity, axial diffusivity, radial diffusivity maps
FDT: FMRIB’s Diffusion Toolbox
FSL: FMRIB Software Library
G: general psychopathology
GAF: Global Assessment of Functioning
JCDSS: Japanese version of the Calgary Depression Scale for Schizophrenia
MANCOVA: multivariate analysis of covariance
MR: magnetic resonance
N: negative symptoms
NMDA receptor: N-Methyl-D-aspartate-type glutamate receptor
P: positive symptoms
PANSS: Positive and Negative Syndrome Scale
PCB: placebo
RM-ANCOVA: repeated-measures of analysis of covariance
ROI: region of interest
SANS: Scale for the Assessment of Negative Symptoms
SC: symbolic coding
TBSS: Tract based spatial statistics
TM: token motor task
TOL: tower of London
VFC: verbal fluency of category
VFL: verbal fluency of letter
VM: verbal memory

## References

[1] Javitt DC, Balla A, Burch S, Suckow R, Xie S, Sershen H (2004). Reversal of phencyclidine-induced dopaminergic dysregulation by N-methyl-D-aspartate receptor/glycine-site agonists. Neuropsychopharmacology.

[2] Nishikawa T, Tanii Y, Umino A, Hashimoto A, Hata N, Takashima M, Shirayama Y, Takahashi K (1991). Phencyclidine, NMDA receptor and schizophrenia. Yakubutsu Seishin Kodo.

[3] Javitt DC (2004). Glutamate as a therapeutic target in psychiatric disorders. Mol Psychiatry.

[4] Tsai GE, Yang P, Chang YC, Chong MY (2006). D-alanine added to antipsychotics for the treatment of schizophrenia. Biol Psychiatry.

[5] Hood WF, Compton RP, Monahan JB (1989). D-cycloserine: a ligand for the N-methyl-D-aspartate coupled glycine receptor has partial agonist characteristics. Neurosci Lett.

[6] Goff DC, Tsai G, Manoach DS, Coyle JT (1995). Dose-finding trial of D-cycloserine added to neuroleptics for negative symptoms in schizophrenia. Am J Psychiatry.

[7] Goff DC, Tsai G, Manoach DS, Flood J, Darby DG, Coyle JT (1996). D-cycloserine added to clozapine for patients with schizophrenia. Am J Psychiatry.

[8] Goff DC, Henderson DC, Evins AE, Amico E (1999). A placebo-controlled crossover trial of D-cycloserine added to clozapine in patients with schizophrenia. Biol Psychiatry.

[9] Goff DC, Tsai G, Levitt J, Amico E, Manoach D, Schoenfeld DA, Hayden DL, McCarley R, Coyle JT (1999). A placebo-controlled trial of D-cycloserine added to conventional neuroleptics in patients with schizophrenia. Arch Gen Psychiatry.

[10] Goff DC, Herz L, Posever T, Shih V, Tsai G, Henderson DC, Freudenreich O, Evins AE, Yovel I, Zhang H (2005). A six-month, placebo-controlled trial of D-cycloserine co-administered with conventional antipsychotics in schizophrenia patients. Psychopharmacology.

[11] Goff DC, Cather C, Gottlieb JD, Evins AE, Walsh J, Raeke L, Otto MW, Schoenfeld D, Green MF (2008). Once-weekly D-cycloserine effects on negative symptoms and cognition in schizophrenia: an exploratory study. Schizophr Res.

[12] van Berckel BN, Hijman R, van der Linden JA, Westenberg HG, van Ree JM, Kahn RS (1996). Efficacy and tolerance of D-cycloserine in drug-free schizophrenic patients. Biol Psychiatry.

[13] van Berckel BN, Evenblij CN, van Loon BJ, Maas MF, van der Geld MA, Wynne HJ, van Ree JM, Kahn RS (1999). D-cycloserine increases positive symptoms in chronic schizophrenic patients when administered in addition to antipsychotics: a double-blind, parallel, placebo-controlled study. Neuropsychopharmacology.

[14] Heresco-Levy U, Javitt DC, Ermilov M, Silipo G, Shimoni J (1998). Double-blind, placebo-controlled, crossover trial of D-cycloserine adjuvant therapy for treatment-resistant schizophrenia. Int J Neuropsychopharmacol.

[15] Heresco-Levy U, Ermilov M, Shimoni J, Shapira B, Silipo G, Javitt DC (2002). Placebo-controlled trial of D-cycloserine added to conventional neuroleptics, olanzapine, or risperidone in schizophrenia. Am J Psychiatry.

[16] Evins AE, Amico E, Posever TA, Toker R, Goff DC (2002). D-Cycloserine added to risperidone in patients with primary negative symptoms of schizophrenia. Schizophr Res.

[17] Cascella NG, Macciardi F, Cavallini C, Smeraldi E (1994). D-cycloserine adjuvant therapy to conventional neuroleptic treatment in schizophrenia: an open-label study. J Neural Transm Gen Sect.

[18] Rosse RB, Fay-McCarthy M, Kendrick K, Davis RE, Deutsch SI (1996). D-cycloserine adjuvant therapy to molindone in the treatment of schizophrenia. Clin Neuropharmacol.

[19] Duncan EJ, Szilagyi S, Schwartz MP, Bugarski-Kirola D, Kunzova A, Negi S, Stephanides M, Efferen TR, Angrist B, Peselow E (2004). Effects of D-cycloserine on negative symptoms in schizophrenia. Schizophr Res.

[20] Buchanan RW, Javitt DC, Marder SR, Schooler NR, Gold JM, McMahon RP, Heresco-Levy U, Carpenter WT (2007). The cognitive and negative symptoms in schizophrenia trial (CONSIST): the efficacy of glutamatergic agents for negative symptoms and cognitive impairments. Am J Psychiatry.

[21] Veru F, Jordan G, Joober R, Malla A, Iyer S (2016). Adolescent vs. adult onset of a first episode psychosis: impact on remission of positive and negative symptoms. Schizophr Res.

[22] Stentebjerg-Olesen M, Pagsberg AK, Fink-Jensen A, Correll CU, Jeppesen P (2016). Clinical characteristics and predictors of outcome of schizophrenia-Spectrum psychosis in children and adolescents: a systematic review. J Child Adolesc Psychopharmacol.

[23] Dazzan P (2014). Neuroimaging biomarkers to predict treatment response in schizophrenia: the end of 30 years of solitude?. Dialogues Clin Neurosci.

[24] Smith SM, Jenkinson M, Johansen-Berg H, Rueckert D, Nichols TE, Mackay CE, Watkins KE, Ciccarelli O, Cader MZ, Matthews PM (2006). Tract-based spatial statistics: voxelwise analysis of multi-subject diffusion data. NeuroImage.

[25] Clemmensen L, Vernal DL, Steinhausen HC (2012). A systematic review of the long-term outcome of early onset schizophrenia. BMC Psychiatry.

[26] Takahashi A, Lee RX, Iwasato T, Itohara S, Arima H, Bettler B, Miczek KA, Koide T (2015). Glutamate input in the dorsal raphe nucleus as a determinant of escalated aggression in male mice. J Neurosci.

[27] Tsai GE, Lin PY (2010). Strategies to enhance N-methyl-D-aspartate receptor-mediated neurotransmission in schizophrenia, a critical review and meta-analysis. Curr Pharm Des.

[28] Quartermain D, Mower J, Rafferty MF, Herting RL, Lanthorn TH (1994). Acute but not chronic activation of the NMDA-coupled glycine receptor with D-cycloserine facilitates learning and retention. Eur J Pharmacol.

[29] Parnas AS, Weber M, Richardson R (2005). Effects of multiple exposures to D-cycloserine on extinction of conditioned fear in rats. Neurobiol Learn Mem.

[30] Nong Y, Huang YQ, Ju W, Kalia LV, Ahmadian G, Wang YT, Salter MW (2003). Glycine binding primes NMDA receptor internalization. Nature.

[31] Uezato A, Yamamoto N, Iwayama Y, Hiraoka S, Hiraaki E, Umino A, Haramo E, Umino M, Yoshikawa T, Nishikawa T (2015). Reduced cortical expression of a newly identified splicing variant of the DLG1 gene in patients with early-onset schizophrenia. Transl Psychiatry.

[32] Zhuo C, Liu M, Wang L, Tian H, Tang J (2016). Diffusion tensor MR imaging evaluation of Callosal abnormalities in schizophrenia: a meta-analysis. PLoS One.

[33] Fujiwara H, Namiki C, Hirao K, Miyata J, Shimizu M, Fukuyama H, Sawamoto N, Hayashi T, Murai T (2007). Anterior and posterior cingulum abnormalities and their association with psychopathology in schizophrenia: a diffusion tensor imaging study. Schizophr Res.

[34] Bopp MH, Zollner R, Jansen A, Dietsche B, Krug A, Kircher TT. White matter integrity and symptom dimensions of schizophrenia: a diffusion tensor imaging study. Schizophr Res. 2016; 10.1016/j.schres.2016.11.045 28012640

[35] Lee SJ, Kim B, Oh D, Kim MK, Kim KH, Bang SY, Choi TK, Lee SH (2016). White matter alterations associated with suicide in patients with schizophrenia or schizophreniform disorder. Psychiatry Res.

[36] Takei K, Yamasue H, Abe O, Yamada H, Inoue H, Suga M, Sekita K, Sasaki H, Rogers M, Aoki S (2008). Disrupted integrity of the fornix is associated with impaired memory organization in schizophrenia. Schizophr Res.

[37] Ozcelik-Eroglu E, Ertugrul A, Oguz KK, Has AC, Karahan S, Yazici MK (2014). Effect of clozapine on white matter integrity in patients with schizophrenia: a diffusion tensor imaging study. Psychiatry Res.

[38] Mamah D, Conturo TE, Harms MP, Akbudak E, Wang L, McMichael AR, Gado MH, Barch DM, Csernansky JG (2010). Anterior thalamic radiation integrity in schizophrenia: a diffusion-tensor imaging study. Psychiatry Res.

